# From electricity to vitality: the emerging use of piezoelectric materials in tissue regeneration

**DOI:** 10.1093/burnst/tkae013

**Published:** 2024-07-02

**Authors:** Yifan Wu, Junwu Zou, Kai Tang, Ying Xia, Xixi Wang, Lili Song, Jinhai Wang, Kai Wang, Zhihong Wang

**Affiliations:** College of Life Sciences, Tiangong University, Binshuixi Road, Xiqing District, Tianjin 300387, China; College of Life Sciences, Key Laboratory of Bioactive Materials (Ministry of Education), State Key Laboratory of Medicinal Chemical Biology, Nankai University, Weijin Road, Nankai District, Tianjin 300071, China; College of Life Sciences, Tiangong University, Binshuixi Road, Xiqing District, Tianjin 300387, China; State Key Laboratory of Cardiovascular Disease, Department of Cardiovascular Surgery, National Center for Cardiovascular Diseases, Chinese Academy of Medical Sciences, Peking Union Medical College, Fuwai Hospital, Beilishi Road, Xicheng District, Beijing 100037, China; College of Life Sciences, Tiangong University, Binshuixi Road, Xiqing District, Tianjin 300387, China; College of Life Sciences, Tiangong University, Binshuixi Road, Xiqing District, Tianjin 300387, China; Tianjin Key Laboratory of Biomaterial Research, Institute of Biomedical Engineering, Chinese Academy of Medical Sciences and Peking Union Medical College, Baidi Road, Nankai District, Tianjin 300192, China; College of Life Sciences, Tiangong University, Binshuixi Road, Xiqing District, Tianjin 300387, China; Tianjin Key Laboratory of Biomaterial Research, Institute of Biomedical Engineering, Chinese Academy of Medical Sciences and Peking Union Medical College, Baidi Road, Nankai District, Tianjin 300192, China; College of Life Sciences, Tiangong University, Binshuixi Road, Xiqing District, Tianjin 300387, China; College of Life Sciences, Key Laboratory of Bioactive Materials (Ministry of Education), State Key Laboratory of Medicinal Chemical Biology, Nankai University, Weijin Road, Nankai District, Tianjin 300071, China; Institute of Transplant Medicine, School of Medicine, Nankai University, Weijin Road, Nankai District, Tianjin 300071, China

**Keywords:** Smart materials, Piezoelectric, Tissue engineering, Scaffold, Regenerative medicine electrical stimulation

## Abstract

The unique ability of piezoelectric materials to generate electricity spontaneously has attracted widespread interest in the medical field. In addition to the ability to convert mechanical stress into electrical energy, piezoelectric materials offer the advantages of high sensitivity, stability, accuracy and low power consumption. Because of these characteristics, they are widely applied in devices such as sensors, controllers and actuators. However, piezoelectric materials also show great potential for the medical manufacturing of artificial organs and for tissue regeneration and repair applications. For example, the use of piezoelectric materials in cochlear implants, cardiac pacemakers and other equipment may help to restore body function. Moreover, recent studies have shown that electrical signals play key roles in promoting tissue regeneration. In this context, the application of electrical signals generated by piezoelectric materials in processes such as bone healing, nerve regeneration and skin repair has become a prospective strategy. By mimicking the natural bioelectrical environment, piezoelectric materials can stimulate cell proliferation, differentiation and connection, thereby accelerating the process of self-repair in the body. However, many challenges remain to be overcome before these concepts can be applied in clinical practice, including material selection, biocompatibility and equipment design. On the basis of the principle of electrical signal regulation, this article reviews the definition, mechanism of action, classification, preparation and current biomedical applications of piezoelectric materials and discusses opportunities and challenges for their future clinical translation.

Highlights

- The biological effects of endogenous electrical signals *in vivo* are briefly described.
- The piezoelectric effect and the principle of piezoelectric generation are described.
- The main classifications of piezoelectric materials and typical piezoelectric materials are presented.
- The role of piezoelectric materials in the processes of tissue regeneration and organ repair is discussed.

## Background

The deleterious effects of aging, illness, and severe tissue and organ damage due to accidents pose significant threats to human health. Traditional surgical and pharmaceutical interventions have been largely inadequate for the repair and functional reconstruction of damaged areas. Accordingly, researchers have employed tissue engineering techniques to construct biologically active scaffold materials that can promote tissue regeneration at the site of injury [1–4]. Given that most human tissues exhibit piezoelectric properties and that electrical signals play crucial roles in regulating cellular behavior and promoting tissue regeneration, piezoelectric materials that can spontaneously generate electrical signals have attracted considerable interest as ‘electrically active’ biomaterials.

Piezoelectric materials possess the extraordinary capability of converting mechanical energy into electrical energy and vice versa without the need for external electrical stimulation devices. This exceptional property is an important advantage over conventional materials, making piezoelectric materials promising candidates for diverse applications in biomedical engineering. The generated electrical signals can improve the physiological electrical environment, promoting tissue repair and regeneration. Piezoelectric materials have emerged as a novel class of smart materials that are being increasingly utilized in various areas, such as medical sensors, energy harvesting, and drug control and release systems [5–10].

Piezoelectric materials have been found to play critical roles in tissue repair, particularly in bone healing and nerve regeneration [11]. Researchers have utilized 3D printing, electrostatic spinning and other technologies to make piezoelectric materials into fibrous scaffolds that can match the size of damaged tissues. Following implantation into the injured site, these scaffolds can generate electrical signals in response to external mechanical stresses, which subsequently activate relevant signaling pathways and modulate cellular behaviors such as adhesion, migration, proliferation and differentiation [12], thereby promoting tissue regeneration. For instance, in bone tissue engineering, piezoelectric scaffold materials can simulate the physiological and microelectrical environment of bone tissue to facilitate osteogenesis and promote the repair of bone tissue defects [13–16]. In neural tissue engineering, electrical pulses can stimulate targeted nerve axon growth and promote nerve tissue repair following injury [17].

**Figure 1:**
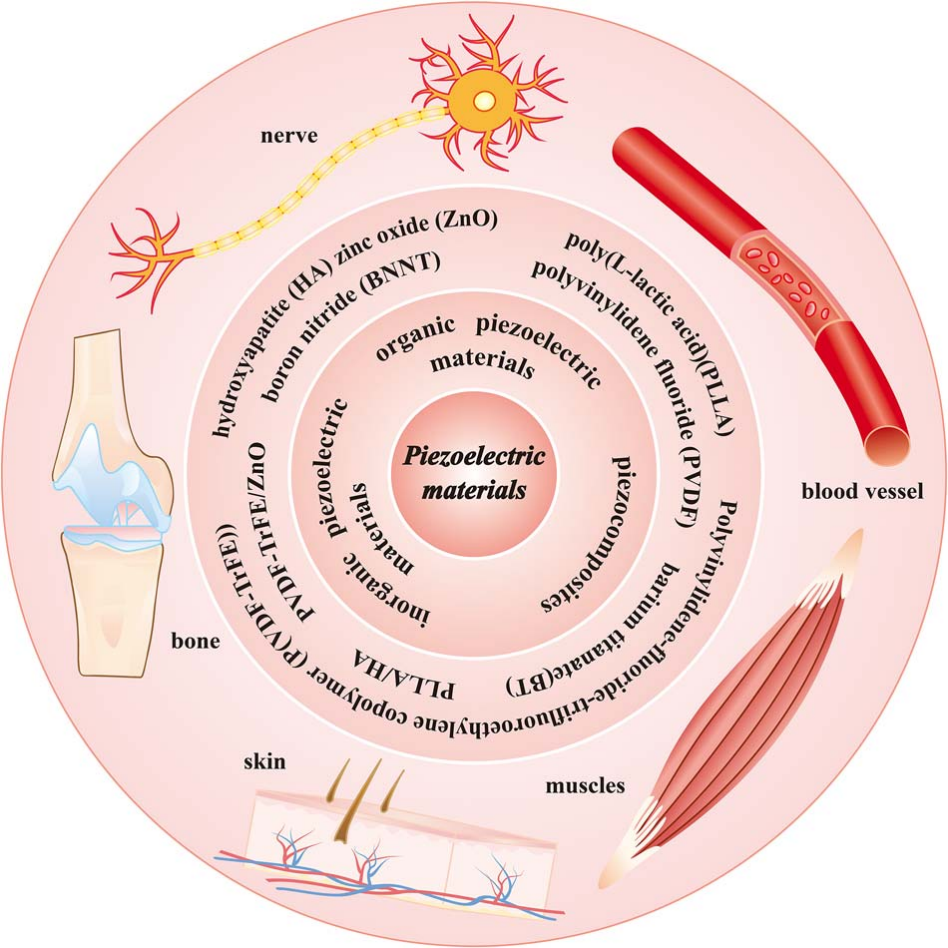
Application of piezoelectric materials in tissue engineering

Although research in this field is still in an early stage of development, the application of piezoelectric materials in tissue engineering is promising and is expected to revolutionize regenerative medicine and biomedical devices. There are many reports of the use of piezoelectric materials in sensors and drug delivery. As research in this area expands, there is anticipation of groundbreaking advancements in harnessing piezoelectric materials for tissue engineering and integrating them with current biomedical technologies, thereby driving innovation in healthcare. This paper provides an overview of the piezoelectric properties of various materials and highlights research on their applications in diverse tissue engineering fields, as illustrated in Figure 1.

## Review

### Endogenous bioelectricity and the piezoelectric effect

Bioelectricity is integral to living systems, and endogenous electric fields affect cell behavior, intercellular communication and tissue healing [18,19]. In bone tissue, endogenous bioelectricity regulates apatite precipitation and calcification through piezoelectric effects. Calcium phosphate is a key inorganic salt in bone tissue and exists in the forms of crystalline hydroxyapatite (HA) and amorphous calcium phosphate [20]. There is a potential difference between the inside and outside of the cell, known as the membrane potential [21]. By regulating the activity of ion channels in the cell membrane, endogenous bioelectricity controls changes in membrane potential and consequently in ion concentrations inside and outside the cell, affecting ion flow and electrical signal transmission between cells. Calcium ion is a key factor in regulating bone cell function [22]. The open state of calcium-ion channels is regulated by the endogenous electric field and in turn regulates ion flow, thus affecting the proliferation, differentiation and bone matrix synthesis of osteocytes [23].

**Figure 2:**
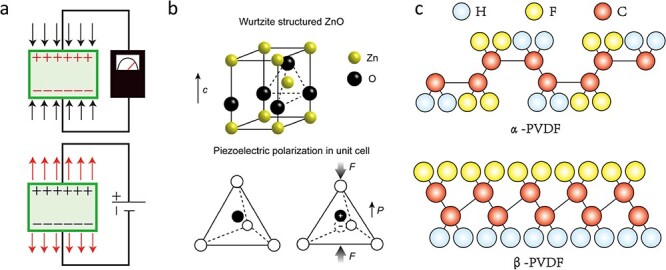
Piezoelectricity and the piezotronic effect. (**a**) Positive piezoelectric effect and inverse piezoelectric effect. (**b**) Piezoelectricity in wurtzite semiconductors. Atomic model of the wurtzite-structured ZnO and the origin of piezoelectricity. (Reprinted with permission from ref. [24] © 2021 Elsevier B.V.) (**c**) The α and β primary polymorphic crystalline phases of PVDF. *ZnO* zinc oxide, *PVDF* polyvinylidene fluoride

The generation of electrical energy on the surface of a material due to an applied force is referred to as the direct piezoelectric effect or, alternatively, the positive piezoelectric effect. Similarly, the mechanical deformation of a material in response to an applied electric field is known as the inverse piezoelectric effect, as illustrated in Figure 2a.

Piezoelectric effects have been observed in a diverse range of materials, including both inorganic and organic polymeric materials. In inorganic materials, piezoelectricity arises from a non-centrosymmetric crystal structure, which generates an electric dipole moment within the stressed crystal. For example, in the ZnO crystal structure, in the absence of stress or strain, Zn^2+^ and O^2−^ ions are tetrahedrally coordinated, resulting in overlapping centers of positive and negative charges without any polarization. However, under tensile or compressive stresses, the relative displacement of Zn^2+^ and O^2−^ leads to the generation of a dipole moment (Figure 2b) [24]. The piezoelectricity of organic materials originates from the orientation and arrangement of molecular dipoles. For instance, the piezoelectric property of polyvinylidene fluoride (PVDF) exists due to the difference in electronegativity between F and H atoms, which can produce a dipole moment. The α-phase does not exhibit piezoelectricity due to the net cancellation of the dipole moments formed in opposite directions. In contrast, the β-phase displays the highest piezoelectricity owing to the parallel orientation of the dipole moment, resulting in the highest net electric dipole moment (Figure 2c) [25].

The piezoelectric properties of a material can be increased by means of polarization in a high external electric field. In other words, polarization can improve the piezoelectric properties of a material. Thermal energy at high temperature can increase the mobility of molecular chains to promote polarization through thermal polarization, the direct application of an electric field on the surface of materials can achieve contact polarization and high-potential corona discharge can generate ions near materials through noncontact polarization: these ions are charged in the electric field, leading to surface polarization of the material [26]. These polarization methods help to increase the efficiency of biomedical energy harvesting applications of piezoelectric materials.

### Electroactive biomaterials

#### Piezoelectric materials

Piezoelectric materials are divided into three main categories according to their composition: inorganic piezoelectric materials, organic piezoelectric materials (including natural and synthetic biopolymers) and piezocomposites (Table 1).

**Table 1:** Classification of piezoelectric materials

**Piezoelectric material**		**Characteristics**	**Piezoelectric device**	**Piezoelectric coefficient (pC/N)**	**Applications**	**Refs.**
Inorganic piezoelectric material		Strong piezoelectric effect, Long-term stability, strong rigidity	BaTiO_3_, BT	242	Bone, nerve	[6,27]
			HA	/	Bone	[28]
			ZnO	12.4	Skin, muscle, cardiovascular	[29–31]
			BNNT	0.3	Bone	[32,33]
Organic piezoelectric materials	Natural organic piezoelectric materials	Softness and flexibility, good biocompatibility, relatively weak piezoelectric effect	Cellulose	0.2	/	[34]
			Chitosan	0.2–1.5	/	[35]
			Glycine	/	/	[36]
			Diphenylalanine	/	/	[37]
			Collagen	0.2–2.0	/	[17]
			Silk fibroin	/	Skin	[38]
	Synthetic organic piezoelectric materials		PVDF	24–34	Bone, nerve, skin, muscle	[39–43]
			P(VDF-TrFE)	38	Bone, nerve, cardiovascular	[31,43–45]
			PLLA	5–15	Bone, nerve, cardiovascular	[46–50]
			PHB	1.6–2.0	Bone	[51,52]
Piezocomposites		Pliability and good piezoelectric effect, customizability, complex preparation, unstable performance	P(VDF-TrFE)/BT	9.21	Bone, nerve	[44,53]
			P(VDF-TrFE)/ZnO	/	Cardiovascular	[31]
			PLLA/HA	/	Bone	[54]
			PHB/HA	/	Bone	[55]

*BT* barium titanate, *HA* hydroxyapatite, *ZnO* zinc oxide, *BNNT* boron nitrate, *PVDF* polyvinylidene fluoride, *P(VDF-TrFE)* polyvinylidene-fluoride-trifluoroethylene, *PLLA* poly(L-lactic acid), *PHB* polyhydroxybutyrate

##### Inorganic piezoelectric materials

Piezoelectric quartz crystal is a representative single-crystal piezoelectric material composed predominantly of silicon dioxide, also known as crystal [56]. It was discovered by the Curie brothers in 1880 [57] and initiated the development of piezoelectric materials.

Piezoelectric ceramics are polycrystalline materials [58] in which each individual grain forms a small region of spontaneous polarization in the same direction, i.e. an electric domain. Owing to their polycrystalline structure, piezoelectric ceramics exhibit a significantly higher piezoelectric coefficient than quartz crystals and possess advantageous mechanical properties. The first piezoelectric ceramic material used was barium titanate (BaTiO_3_, BT), due to its high piezoelectric coefficient [59,60]. BT is inexpensive, simple and easy to prepare [61] and can exert desirable effects on cell proliferation and differentiation [62–64]. In addition, BT nanoparticles can self-assemble in the tumor microenvironment in response to pH changes, which can cause mechanical damage to tumor cells, facilitating effective tumor clearance [65]. Another piezoelectric material is potassium sodium niobate (KNN), which possesses a complex orthogonal structure [66,67]. However, most piezoelectric ceramics with high piezoelectric coefficients have some degree of cytotoxicity.

The cytotoxicity of BT can be reduced to some extent by adjusting the composition and dosage of the material for use in tissue engineering and regenerative medicine. Functionalizing piezoelectric materials with antioxidants (e.g. *N*-acetylcysteine) can significantly reduce the cytotoxicity caused by BT nanoparticles and enable the promotion of cell growth and tissue regeneration [68]. The deposition of bioactive coatings, such as HA or collagen, on the BT surface can increase its ability to bind to bone tissue while potentially helping to reduce cytotoxicity [69]. Moreover, HA [70], a new piezoelectric material, has been developed for tissue engineering along with zinc oxide (ZnO) [71] and boron nitrate [72].

HA is the main component of natural bone, but the poor mechanical properties of pure HA limit its utility in load-bearing applications. Researchers have toughened the material by introducing other piezoelectric materials (such as KNN), and domain switching can occur under mechanical loading or electrical stimulation, resulting in changes in the mechanical properties of piezoelectric ceramics [73,74]. Before being used as a piezoelectric material, ZnO was widely studied for tissue engineering applications due to its ability to produce reactive oxygen species [75–77]. In recent years, due to its excellent piezoelectric properties, stable chemical properties, abundant yield in Nature and other advantages, researchers from various backgrounds have taken an interest in researching ZnO, which has become a popular material for biomedical sensors and piezoelectric nanogenerators [78]. The amount of Zn ions released by the nanogenerator does not interfere with cellular activity or trigger an associated inflammatory response, and the nanogenerator itself is biocompatible and does not elicit an inflammatory response [30].

##### Organic piezoelectric materials

###### Synthetic organic piezoelectric materials

Compared with those of piezoelectric ceramics, the piezoelectric coefficients of piezoelectric polymers are relatively low, but the advantages of high flexibility and low stiffness give piezoelectric polymers more flexible machining properties than piezoelectric ceramics and are more suitable for soft tissue regeneration [79]. The most commonly used piezoelectric polymers include PVDF [80] and poly(L-lactic acid) (PLLA) [81].

####### PVDF

**Figure 3:**
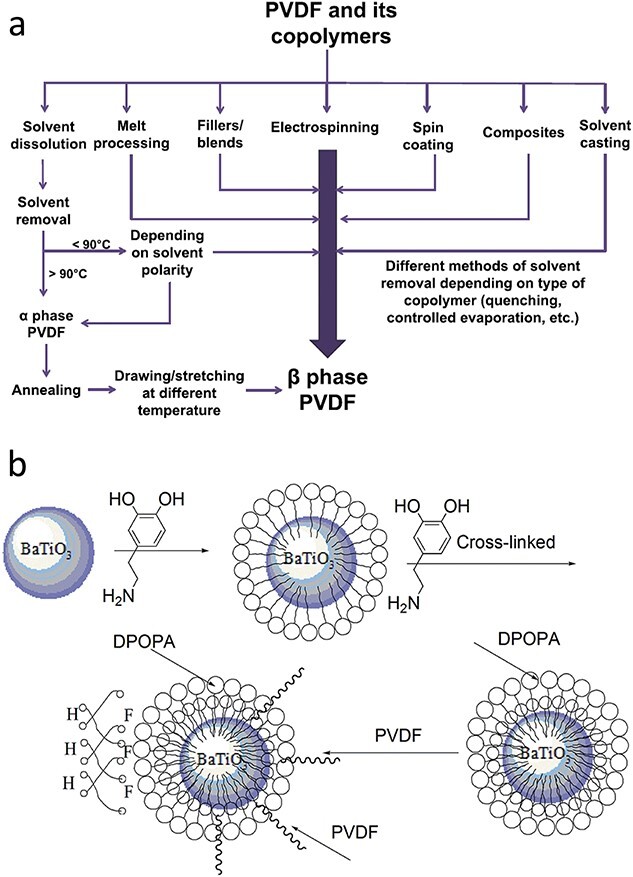
Copolymers and modifications of PVDF. (**a**) Fabrication methods to obtain PVDF and its copolymers (PVDF-TrFE, PVDF-HFP) in β-phase. (Reprinted with permission from ref. [90] © 2018 Elsevier B.V.) (**b**) Schematic illustration of the interface modification between PVDF matrix and BaTiO_3_ fillers. (Reprinted with permission from ref. [134] © 2015 the Royal Society of Chemistry.) *PVDF-TrFE* polyvinylidene-fluoride-trifluoroethylene,* PVDF-HFP* poly(vinylidene fluoride-hexafluoropropylene, *DPOPA* polydopamine

PVDF is the most extensively researched piezoelectric polymer due to its high piezoelectric coefficient [34,82–84], favorable processability and good mechanical strength [85]. It has broad applications in tissue engineering and biosensors [86,87]. PVDF has five primary crystalline forms, α, β, γ, δ and ε, which are dependent on the conformation of its polymer chains [88]. Among these forms, the β-phase content has the strongest effect on the piezoelectric properties. Adjusting parameters such as spinning distance, applied voltage and speed can increase the β-phase content and thus the piezoelectric properties [88,89], as shown in Figure 3 [90]. In addition, further annealing can increase the β-phase orientation, resulting in a higher voltage output [91]. Polyvinylidene-fluoride-trifluoroethylene [P(VDF-TrFE)] is composed of vinylidene fluoride (VDF) and trifluoroethylene (TrFE). P(VDF-TrFE) has an all-*trans* stereochemical configuration due to the extra fluorine atoms in TrFE, which create steric hindrance, and P(VDF-TrFE) is thus a PVDF copolymer with a β-phase [92] that has a higher piezoelectric coefficient than PVDF [93].

####### PLLA and others

Polylactic acid (PLA) is a thermoplastic aliphatic polyester that is biodegradable, bioresorbable and biocompatible. The US Food and Drug Administration (FDA) has approved PLA for use in implantable medical devices, drug delivery vehicles and tissue regeneration scaffolds. PLA exists in three isomeric forms, namely, poly(D-lactic acid) (PDLA), PLLA and racemic poly(DL-lactide) (PDLLA) [94,95]. Among these forms, PLLA exhibits greater chemical stability and biocompatibility than PDLA, and the L-lactic acid generated during its degradation is not harmful to humans [96]. PLLA has two stable thermodynamic conformations, α and α′, which have different piezoelectric properties. Heat treatment enables the interconversion of these two conformations, and Tai *et al*. reported a decrease in the amount of α-phase and an increase in the amount of α′-phase upon heat treatment of PLLA [97]. The transverse piezoelectric voltage output is dependent on the α-phase content, while the longitudinal piezoelectric voltage is correlated with the α′-phase content. Increasing the temperature decreases the longitudinal piezoelectric voltage but increases the transverse piezoelectric voltage [97].

Polyhydroxyalkanoates, another commonly used biodegradable piezoelectric polymer, are aliphatic copolyesters with varying structures that are synthesized by microorganisms [98]. Polyhydroxyalkanoates include polyhydroxybutyrate (PHB) and poly(3-hydroxybutyrate-3-hydroxyvalerate) (PHBV) [99].

###### Natural organic piezoelectric materials

Natural organic piezoelectric materials have been extensively studied in tissue engineering due to their low toxicity and biodegradability.

####### Polysaccharides

Representative polysaccharides include cellulose [100,101] and chitosan [102]. Driven by the demand for environmentally friendly materials in recent years, cellulose and its derivatives have become promising natural materials in the field of piezoelectric energy harvesting due to their abundant yield, renewable material, high sustainability and low cost [103,104]. Cellulose is a linear polysaccharide with glucose as the repeating unit. Because of its high crystallinity, abundant hydroxyl polar groups are arranged along the asymmetric monoclinic and triclinic regions of cellulose, and asymmetric dipoles with piezoelectric activity can easily form [105]. Chitosan is the second largest natural polysaccharide on Earth after cellulose. Like cellulose, chitosan also has piezoelectric properties due to its non-centrosymmetric crystal structure and is used in the field of sensors [106,107].

####### Amino acids

In recent years, amino acid and peptide piezoelectric biomaterials have attracted extensive attention from researchers. Amino acids are the basic building blocks of proteins. Their molecular structure contains an amino group (-NH_2_), a carboxyl group (-COOH) and side chains (R) that distinguish different amino acids. The piezoelectric properties of amino acids are affected by the crystal structure, which determines the ordered arrangement and interaction of the electric dipole moments inside the molecule [108]. These properties enable specific crystal forms of amino acids to exhibit piezoelectric effects. Glycine is the only achiral amino acid and has three phases: α, β and γ. Among them, the γ-phase is the most stable and has a unique helical hydrogen bond network, while the α- and β-phases are stabilized by different lamellar hydrogen bond networks [36]. Different crystalline phases can be interconverted under certain conditions; the α-phase and β-phase may change into the γ-phase when the humidity increases, and the γ-phase can change into the α-phase with increasing temperature [36]. The β- and γ-phases exhibit significant piezoelectricity due to their non-centrosymmetric structure [109,110]. This piezoelectric property makes glycine a potential material for developing piezoelectric sensors and piezoelectric energy harvesting [111–113]. The piezoelectricity of other amino acids involves their chiral symmetry [114]. Examples include DL-alanine [115,116], L-leucine [117], L-tyrosine [118] and isoleucine [119]. The piezoelectric properties of these amino acids demonstrate the potential of natural amino acids as piezoelectric materials for use in biomedical devices. Researchers are exploring the piezoelectric properties of a variety of amino acids to develop new applications in biomedical fields.

####### Peptides

Peptides are short-chain molecules composed of multiple amino acids linked by peptide bonds. Diphenylalanine peptides have been widely studied due to their simple structure and significant piezoelectric properties [37]. Owing to their large molecular size, peptide molecules have high degrees of freedom in space and can spontaneously assemble into complex structures through intermolecular interactions, such as hydrogen bonds, electrostatic interactions and hydrophobic interactions between the specific functional groups of each amino acid unit [120]. Through this self-assembly process, peptides can form ordered structures, and this ordered arrangement can lead to the formation of nanostructures with specific directionality [121]. These ordered nanostructures exhibit piezoelectric properties at the macroscopic level.

####### Protein

In addition to amino acids and peptides, piezoelectric proteins such as collagen have been studied extensively. Proteins are biological macromolecules composed of amino acid polymers connected by peptide bonds. Hundreds of combinations of amino acids can form different structures, and proteins with piezoelectric properties have been identified. Collagen molecules consist of long chains of helical structures that, when stretched or twisted, can cause tiny charge separations, resulting in a piezoelectric effect [122]. Despite the relatively weak piezoelectric properties of these materials, researchers have begun to explore their potential applications in the field of sensors [123]. Silk fibroin (SF) is the main component of silkworm cocoons. SF has not only excellent mechanical properties but also significant piezoelectric properties [124], which makes SF a preferred material in the field of flexible electronic products. Using these characteristics of SF, biodegradable piezoelectric/triboelectric nanogenerators can be produced [125]. These devices can not only convert mechanical energy into electrical energy but also act as pressure sensors to monitor physiological signals for medical monitoring [126,127]. In addition, the biocompatibility and degradability of SF offer new possibilities in the field of tissue engineering and regenerative medicine, especially because of its potential to promote wound healing [38]. Elastin is an extracellular matrix (ECM) that is present mainly in connective tissues, confers elasticity and flexibility on tissues such as skin, blood vessels and lung tissue, and participates in biological signal transmission. Elastin exhibits piezoelectric properties under mechanical stress, such as during the dilation and contraction of blood vessels and the expansion and contraction of lung tissue [128,129]. The piezoelectric charge generated by elastin may be involved in the regulation of the proliferation and migration of vascular smooth muscle cells and in the repair of blood vessels in the context of vascular injury or disease, affecting the morphology and function of blood vessels [128]. The generation of charge by elastin might also play a role in the binding of oxygen to hemoglobin and the regulation of intravascular pressure [129].

For future applications in tissue engineering, the weak piezoelectric properties of piezoelectric proteins can be exploited, but it is often necessary to combine materials or techniques to adjust the piezoelectric properties of materials to meet specific requirements. These natural piezoelectric polymers present promising opportunities for the development of a new generation of tissue engineering piezoelectric materials.

##### Piezocomposites

Piezocomposites combine the advantages of piezoelectric ceramics and piezoelectric polymers, exhibiting both high piezoelectric coefficients and good flexibility. Improving the properties of piezoelectric composites can further promote the application of piezoelectric biomaterials in tissue engineering [130].

The piezoelectric properties of PVDF, the most widely used piezoelectric polymer, can be significantly improved by the addition of nanofillers such as ZnO [131] and BT [132,133]. This improvement occurs because BT nanoparticles increase the content of the β-phase in PVDF (Figure 3b) [134]. Similarly, the uniform dispersion of ZnO nanoparticles in PVDF can increase the β-phase content and thus the piezoelectric output [135]. The introduction of piezoelectric ceramic particles into piezoelectric polymers is of great interest to researchers because it is easy to achieve and effectively improves performance.

Polycaprolactone (PCL) is a semicrystalline polymer that exhibits good flexibility [136]. It is an FDA-approved biomaterial with excellent biocompatibility [137] and easy fabrication and manipulation [138] and has been extensively utilized in tissue engineering [139]. Compared with the traditional piezoelectric polymer PVDF, PCL has the advantage of being biodegradable. It has been reported that PCL-based piezoelectric composites were prepared by blending PCL with BT via the solution method [140]. Due to the addition of BT, the PCL/BT piezoelectric composite exhibits piezoelectric properties, and the PCL/BT piezoelectric composite is more conducive to cell adhesion than was pure PCL. In future work, piezoelectric ceramic particles will be introduced into traditional tissue engineering polymer materials to improve the piezoelectric properties of composite materials, thus forming new intelligent piezoelectric materials for tissue engineering applications [140].

#### Electrically conductive biomaterials

In addition to piezoelectric biomaterials, conductive biomaterials have been identified as electroactive materials with potential applications in tissue engineering [141,142]. Although this review focuses on research progress related to piezoelectric materials, it is worth noting that conductive biomaterials can deliver electrical charges and modulate cellular behavior, which might synergistically enhance the efficacy of electrical stimulation for tissue regeneration when used in combination with piezoelectric biomaterials.

At present, there exist three primary classes of conductive materials: conductive carbon-based materials, metallic materials and conductive polymers.

##### Conductive carbon-based materials

Graphene (GP) and carbon nanotubes are extensively investigated conductive carbon-based biomaterials with broad applications in tissue regeneration engineering research, particularly in the areas of skeletal and neural tissue engineering [143–145]. The unique material structure of GP and its derivatives imparts excellent mechanical properties, biocompatibility and high electrical conductivity [146]. Following treatment with potent oxidizing agents, GP can be converted into graphene oxide (GO), which comprises diverse oxygen-containing functional groups, such as hydroxyl, carboxyl and epoxy groups, and exhibits favorable hydrophilicity [147,148]. The incorporation of various bioactive materials can further enhance the mechanical properties of GO and ameliorate the hydrophobicity of the polymer to enable the design of hydrophilic materials suitable for tissue regeneration [149,150]. Reduced GO (rGO) is synthesized chemically from GO [151]. Carbon nanotubes possess outstanding flexibility, strength, electrical conductivity and hydrophilic properties that increase cellular infiltration, modulate cellular behavior and promote cell growth.

##### Metallic materials

Gold nanoparticles (AuNPs) have several promising properties, such as biocompatibility, easy surface modification, good stability and optical properties, that can be exploited in tissue engineering [152]. The ability of these materials to promote cell growth and proliferation enables them to play a key role in repairing damaged tissues [153]. In particular, the electrical properties of AuNPs can increase the electrical communication between adjacent cardiac cells [154]. Silver nanoparticles (AgNPs) have been shown to regulate the proliferation and differentiation of mesenchymal stem cells (MSCs) in bone regeneration and have attracted the attention of researchers due to their osteoinductive properties [155]. Silver is also widely used in the field of antibacterial nanomaterials. AgNPs can damage the bacterial cell membrane, inhibit enzyme activity, and inhibit ATP production and DNA replication during ion transport to exert antibacterial effects [156,157]. Copper is an essential trace element for the repair of various tissues and organs, such as bone and skin, and can accelerate cell proliferation, stimulate new blood vessel formation and inhibit bacterial growth [158]. In skin tissue engineering, copper plays a key role in the biosynthesis of skin ECM, which promotes the production and stability of various skin proteins and fibrous components, including type I, II and V collagen, elastin and fibrillin [158]. In addition to Au, Ag and Cu, other metals, such as Ti, Mg and Fe, have also been used in tissue engineering [159–162].

##### Conductive polymers

The primary conductive polymers utilized are polypyrrole (PPy), polyaniline (PANi), poly(3,4-ethylenedioxythiophene) and polythiophene [163–168]. PPy has excellent biocompatibility in conjunction with high electrical conductivity and can therefore promote cell adhesion, proliferation, differentiation and tissue regeneration [169,170]. It is extensively employed in the manufacture of biological scaffolds [171]. The brittleness of PPy can be enhanced through blending with other polymers that have superior mechanical properties [172]. Polyurethane (PU) is widely used in tissue engineering due to its biocompatibility, outstanding mechanical strength, flexibility and processability [173]. The combination of PU and PPy in a composite scaffold enables PU to provide suitable mechanical properties for tissue growth, while PPy confers electrical conductivity [174]. Moreover, the blending of PCL and PPy produces a conductive scaffold that promotes osteoblast adhesion, proliferation and differentiation under electrical stimulation [175]. In neural regeneration, PPy significantly promotes axonal and myelin regeneration *in vivo*, and a conductive composite scaffold comprising PPy and filamentous protein (SF) promotes the proliferation and migration of Schwann cells upon electrical stimulation of the scaffold [171]. PANi, which possesses high stability and electrical conductivity and facilitates nanofiber formation, is the second most extensively studied conductive organic polymer after PPy [176,177].

### Application of piezoelectric materials in tissue regeneration

Electrical stimulation has been confirmed to promote specific tissue regeneration and repair [178]. In recent years, piezoelectric brackets have been used to produce electrical impulses and thus stimulate cells, and it has been increasingly shown that exogenous electrical stimulation is a promising option for repairing tissue damage. This innovative approach takes full advantage of the concept of electrobiological interactions and provides a new avenue for developing more effective tissue regeneration strategies.

A variety of human tissues, including bone and nerve tissue, exhibit significant electrical activity and piezoelectric properties [122]. The mechanical deformation of the surrounding environmental materials when cells adhere, migrate or move within the human body produces an electric field. The electric fields generated under stress can affect ion channels on the cell membrane, changing the concentration of ions inside and outside the cell and thereby activating signaling pathways within the cell. For example, the Wnt/β-catenin pathway plays a key role in cell proliferation by influencing the cell cycle to promote division, thus increasing the number of cells [179]. In addition, an electric field can guide cells to migrate in the direction of the electric field, a process called electrotaxis [180]. For example, electrical stimulation can promote the proliferation and migration of vascular endothelial cells, which are essential for the formation of granulation tissue and the vascularization of wounds [181]. In tissue repair and inflammation, the electric field can affect the intracellular PI3K/Akt (Phosphoinositide 3-kinase/Protein kinase B signaling pathway) signaling pathway to promote the polarization of macrophages toward the M2 type (anti-inflammatory type), reduce the inflammatory response and promote tissue repair [182]. In summary, electrobiology has illuminated the complex and subtle mechanisms of interaction between cells and their microenvironment.

In this section, our main focus is research on piezoelectric biomaterials in the field of tissue regeneration, especially in electrosensitive tissues. This line of research integrates electrobiology with biomaterials science and opens up new avenues for the development of more precise and efficient regenerative medicine methods.

#### Bone tissue engineering

In bone tissue engineering, the unique piezoelectric properties of piezoelectric materials are used to mimic the electrical signals generated by bone when subjected to mechanical loads, which are essential for osteoblast proliferation and differentiation and for bone matrix deposition [183–185]. The high voltage coefficient and dielectric properties ensure that the material can efficiently generate and maintain the necessary electric field *in vivo* to promote the formation of new bone. Moreover, the mechanical strength and fatigue life of piezoelectric materials are essential for supporting the skeletal structure and for withstanding the stresses generated by daily activities. The stability of these materials under working conditions, especially in environments that mimic physiological temperature and humidity, guarantees their long-term effectiveness in promoting bone tissue regeneration and repair processes. The application of piezoelectric materials in bone tissue engineering can be optimized by precisely regulating their physical and chemical properties and performance parameters, thereby providing innovative solutions for fracture healing, bone defect repair, and the treatment of osteoarthritis and other diseases.

**Figure 4:**
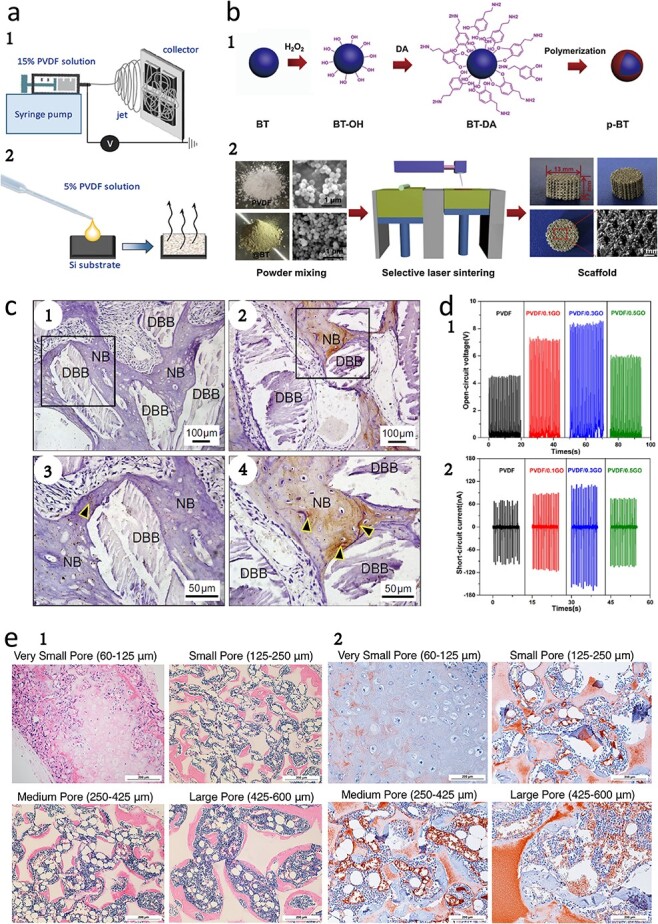
Piezoelectric materials for bone tissue regeneration. (**a**) Schematic representation of the process and photographs of the fabricated scaffolds: (1) electrospinning, (2) drop-casting. (Reprinted with permission from ref. [187] © 2019 the Royal Society of Chemistry.) (**b**) Schematic diagrams of (1) surface modification of BT nanoparticles and (2) fabrication of PVDF/p-BT composite scaffold. (Reprinted with permission from ref. [27] © 2020 Elsevier B.V.) (**c**) Immunohistochemical evaluation of bone regeneration at 12 weeks post-implantation. Representative immunohistochemical staining images for detection of osteocalcin (OCN) expression in deproteinized bovine bone (DBB) granules around nascent bone (NB) tissues covered with polytetrafluoroethylene membranes (1) or BT/P(VDF-TrFE) nanocomposite membranes (2). (3, 4) Enlargements of specific regions of (1) and (2) respectively. Black arrowheads denote the positive expression of OCN. Scale bars:100 μm for (1, 2) and 50 μm for (3, 4). [44]. (**d**) Schematic diagram of the open-circuit voltage (1) and short-circuit current (2) effect of GO concentration on conductivities of scaffold [189]. PVDF/0.1GO, PVDF scaffold with 0.1 wt% GO; PVDF/0.3GO, PVDF scaffold with 0.3 wt% GO; PVDF/0.5GO, PVDF scaffold with 0.5 wt% GO. (**e**) (1) H&E histological analysis after 8 weeks subcutaneous implantation at 100x magnification. Very small-pore scaffold (60–125 mm) contained cartilage with typical morphology in the center of the scaffold. Small-pore (125–250 mm), medium-pore (250–425 mm) and large-pore (425–600 mm) scaffolds supported bone formation on pore walls, shown by pink staining of bone matrix, with bone marrow-like tissue within the pores; n = 3 for each group. Scale bars: 200 μm. (2) CD31 immunohistochemical staining of endothelial layer and visualization of blood cells at 200x magnification as red-brown. Cartilage in very small-pore scaffold is avascular. Blood vessels within small-, medium-, and large-pore scaffold increase in size with increasing pore size. Note that the scaffold also stains red due to the high surface area of the nanofibers; n = 3 for each group. Scale bar: 200 μm. (Reprinted with permission from ref. [192] © 2018 Elsevier B.V.) *BT* baTiO_3_, *DA* dopamine, *BT-OH* hydroxylated BT, *p-BT* polydopamine functionalized BT, *PVDF* polyvinylidene fluoride, *P(VDF-TrFE)* polyvinylidene-fluoride-trifluoroethylene

Szewczyk *et al*. fabricated two types of scaffolds, PVDF(+) and PVDF(−), by controlling the surface potential of PVDF fibers with positive and negative voltage polarities, respectively. The nanofibers generated electrical stimulation to promote collagen production by the cells, and the results showed that the scaffolds fabricated from the PVDF(−) fibers had greater bone regeneration potential than did those fabricated from the PVDF(+) fibers. Collagen can be further mineralized with the help of induced charges, thereby enhancing its function [39]. Ribeiro *et al*. investigated the effect of β-PVDF scaffolds on the differentiation of human adipose-derived stem cells. When human adipose-derived stem cells were cultured on the β-PVDF surface, osteogenic differentiation was detected by quantitative alkaline phosphatase assays [186]. However, the high hydrophobicity of PVDF prevents sufficient cell adhesion and swelling. Kitsara *et al*. successfully fabricated PVDF nanofiber scaffolds with excellent high-voltage electrical properties using electrospinning technology by treating the PVDF surface with oxygen plasma to increase the hydrophilicity of the material, as shown in Figure 4a [187]. These scaffolds not only improved cell adhesion and migration but also promoted cell proliferation and differentiation. In these experiments, human osteosarcoma cells (Saos-2) exhibited excellent growth in the scaffolds, and scanning electron microscopy images revealed that the intracellular calcium concentration was instantaneously changed because the activation of calcium-ion channels and other ion channels in many cells plays important roles in signal transduction [23]. This signaling mechanism significantly promotes osteoblast proliferation.

The piezoelectric properties of PVDF can be increased by doping with various materials, such as BT or GO. Shuai *et al*. doped BT nanoparticles into PVDF scaffolds (Figure 4b) and observed a uniform distribution of BT in the PVDF matrix, a substantial increase in β-phase content, a significant increase in mechanical strength and an increase in output voltage, which provided a stable supporting environment for osteoclast adhesion [27]. Bai *et al*. developed a P(VDF-TrFE)/BT nanocomposite membrane (Figure 4c) with a piezoelectric coefficient similar to that of human bone, which increased neovascularization and significantly increased the rate of bone induction [44]. Saburi *et al*. prepared PVDF/GO nanofiber scaffolds by electrospinning and cultured pluripotent stem cells on PVDF/GO scaffolds. The results showed that the expression levels of Runx2, osteocalcin and osteopontin were significantly greater in these cells than in cells cultured with PVDF alone. This result indicates that PVDF/GO nanofibers can be used as suitable bioimplants for bone tissue engineering applications [188]. Shuai *et al*. fabricated PVDF/GO scaffolds via selective laser sintering and demonstrated that hydrogen bonding interactions between the fluorine group of PVDF and the carbonyl group of GO induced the transition from the α- to the β-phase of PVDF, thereby increasing the electrical output (Figure 4d) [189]. Compared with electrostatic spinning, selective laser sintering enables more precise control of the external shape of the scaffold to meet the individual requirements of bone implants. Genchi *et al*. prepared P(VDF-TrFE)/boron nitrate piezoelectric films and observed increased osteogenic differentiation of cells grown on the material surface under ultrasonic stimulation [190]. Pereira *et al*. mixed PVDF and P(VDF-TrFE) with corn starch. The porous structure of the material, which includes a mixture of starch and cellulose, can support tissue growth, as indicated by an elastic modulus similar to that of cancellous bone. Transplantation studies in male Wistar rats demonstrated that the material adapted well to the cancellous bone environment [191].

PLLA, a biodegradable piezoelectric material, has gained increasing attention in bone tissue engineering [46]. Gupte *et al*. prepared PLLA scaffolds with different pore sizes and reported that small pore sizes (125–250 mm) were more favorable than large pore sizes (425–600 mm) for chondrogenic differentiation and cartilage formation in human bone marrow MSCs (Figure 4e). The authors also demonstrated that the stent pore size can affect bone tissue vascularization [192]. Lai *et al*. demonstrated that the degradation and piezoelectricity of PLLA/rGO/polydopamine (PDA) piezoelectric scaffolds promoted the proliferation of chondrogenic pre-ATDC5 cells into chondrocytes because the addition of rGO affected the electrical output. PDA promoted chondrocyte adhesion and ensured the stability of the cell growth environment [193].

HA, because its chemical composition and structural characteristics are similar to those of bone, displays osteoinductive activity and is extensively used in bone tissue engineering. However, due to the low compressive strength of HA, composite piezoelectric materials comprising combinations of polymers such as PCL, PLA and PHB [28] have been developed. de Siqueira *et al*. fabricated PLLA/PCL/HA nanofibers by electrospinning, and the PLLA/PCL fiber mesh was combined with aggregates of nanophase HA [194]. Compared with those of the nonoriented fibers, the Young’s modulus and tensile strength of the oriented fibers were 15 times and 8 times higher, respectively, and the number of colony-forming units in the tissue grown on the oriented fibers was lower. These results demonstrated the ability of these materials to support osteoblast adhesion and proliferation while promoting cell metabolic activity. Poly(lactic acid-glycolic acid) (PLGA), a copolymer of lactic acid and glycolic acid approved by the FDA for therapeutic devices owing to its biodegradability and biocompatibility [195], was utilized by Babilotte *et al*. in the preparation of PLGA/HA composite bioscaffolds through 3D printing technology [196]. Controlled experiments verified the biocompatibility of the scaffold with human bone marrow stromal cells and human adipose stem cells, and the presence of HA improved its overall bioactivity in bone tissue engineering applications. Fazeli *et al*. doped bioactive glass (BG) into PCL/HA scaffolds using 3D printing technology to fabricate three-component PCL/HA/BG scaffolds for culturing human adipose stem cells [197]. BG, an inorganic bioactive material commonly used in bone tissue engineering [198], can offset the inherent hydrophobicity and poor cell adhesion of PCL, and the surface modification of 3D-printed scaffolds effectively promotes stem cell proliferation and osteogenic differentiation.

In addition to PCL and PLLA, PHB can also be utilized with HA to form a composite material. As a member of the polyhydroxyalkanoate family, PHB is characterized by non-biotoxic degradation products that can be easily metabolized by organisms, making it a highly biocompatible and osteoinductive material that has been used extensively in biomedical and tissue engineering applications [51,199]. Degli Esposti *et al*. obtained porous PHB by thermally induced phase-separation and generated HA particles in PHB using *in situ* synthetic fillers [55]. The composites synthesized *in situ* had higher porosity and did not show degradation of the polymer matrix even at high packing amounts. As a scaffold for bone tissue regeneration, the PHB/HA composite can effectively maintain the adhesion and proliferation of MC3T3-E1 mouse osteoblast precursor cells. Additionally, Volkov *et al*. reported the successful filling of a PHB/HA hybrid scaffold with alginate hydrogels containing MSCs [200]. The PHB/HA scaffolds were capable of supporting MSC growth and promoting the osteogenic differentiation of cells *in vitro*, as evidenced by increased alkaline phosphatase activity and the expression of phenotypic markers such as CD45, which is widely used in MSC phenotypic analysis. Mohan et al. synthesized PHB/OMMT nanocomposites using organic modified montmorillonite (OMMT) as filler. By adding OMMT, the thermal stability and working temperature of the polymer are improved, while the formation of surface pores is promoted. Morphological analysis showed that the nano-composite had better performance than pure PHB, especially in terms of porosity, non-immunogenicity and biocompatibility, indicating that it has potential application prospects in bone tissue engineering and other fields.

PANi has also shown potential for use in bone tissue engineering [201]. In a study conducted by Mirzaei *et al*., PVDF/PANi composite scaffolds were fabricated using electrostatic spinning, and dental pulp stem cells cultured on the scaffolds were treated with very low-frequency pulsed electromagnetic fields (PEMFs) [202]. The results indicated that PEMF treatment stimulated cell adhesion and protein adsorption, increasing cell viability. Furthermore, the presence of conductive polymers, such as PANi, increased the positive effect of PEMFs on osteoblast differentiation. In another study by Chernozem *et al*., PHB was compounded with PCL to produce a material with piezoelectric properties and good hydrophilicity, allowing osteoblasts to adhere and spread on the scaffold surface [203].

#### Neural tissue engineering

In the field of neural tissue engineering, piezoelectric materials are used to simulate the electrophysiological environment of nerve cells in their natural state, as electrical signals are essential for communication between neurons and other cells [204,205]. These materials can promote the growth of nerve cells, axon extension and synapse formation by generating electrical signals that are synchronized with nerve activity to complete the reconstruction of neural networks, which is particularly important for nerve regeneration after injury. In addition, the structure of the scaffold itself has an important effect on the nerve regeneration process. Fibrous scaffolds with an oriented structure have an advantage over those without an oriented structure because highly aligned fibers help guide axon growth and extension [206,207]. This directional structure provides guidance for neuronal connections and facilitates important connections between nerve cells.

**Figure 5:**
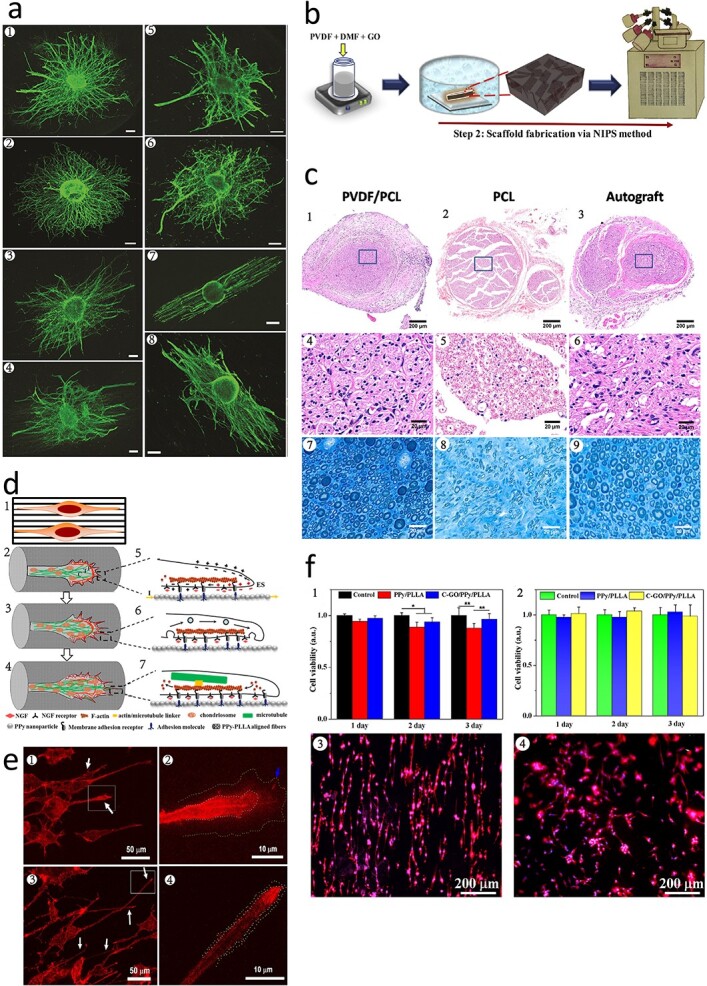
Piezoelectric materials for neural tissue regeneration. (**a**) Confocal fluorescent images of DRG stained with phalloidin (actin) on nano-sized as-spun and annealed random (1 and 2) and aligned (3 and 4) PVDF–TrFE; and micron-sized as-spun and annealed random (5 and 6) and aligned (7 and 8) PVDF–TrFE scaffolds. (Reprinted with permission from ref. [45] © 2011 Elsevier B.V.) (**b**) Schematic diagram showing the immersion precipitation approach followed by the freeze drying method to develop porous PVDF-GO nanocomposite scaffold. [209]. (**c**) Morphological and ultrastructural characterization of the regenerated nerves. (1–6) H&E staining images of the sciatic nerves. Scale bars: 200 or 20 μm. (7–9) TB staining images of the sciatic nerves. Scale bars 20 μm. (Reprinted with permission from ref. [210] © 2020 Elsevier B.V.) (**d**) Schematic diagram of axon elongation from PC12 cells on aligned fibers after differentiation (1), the change of growth cone (2–4) and their inner change of filopodia during the elongation (5–7). (Reprinted with permission from ref. [212] © 2016 the American Chemical Society.) (**e**) Confocal immunofluorescence images of neurites from PC12 on ECM-CFF without electrical stimulation (ES) (1, 2) and with ES (3, 4) of 1 day. (Reprinted with permission from ref. [213] © 2017 Elsevier B.V.) (**f**) MTT results for L929 (1) and PC 12 (2) cells cultured on two films, respectively. Images of stained PC 12 cells cultured onto C-GO/PPy/PLLA film with ES (3) and without ES (4). (Reprinted with permission from ref. [214] © 2019 John Wiley & Sons, Inc.) *DRG* dorsal root ganglion, *H&E* hematoxylin-eosin, *TB *Toluidine blue, *ECM-CFF* ECM-coated con-ductive fibers-film, *MTT* 3-(4,5)-dimethylthiahiazo (-z-y1)-3,5-di-phenytetrazoliumromide, *C-GO* carboxylic graphene oxide, *PPy* polypyrrole, *PLLA* poly-L-lactic acid, *PVDF* polyvinylidene fluoride, *GO* graphene oxide, *NIPS* non-solvent induced phase separation, *PCL* polycaprolactone

PVDF has been found to promote neurite elongation in rat dorsal root ganglion cells, stimulate the secretion of neurotrophic factors by Schwann cells and induce the differentiation of neural stem cells into neurons [40]. Lee *et al*. developed annealed scaffolds with oriented structures using P(VDF-TrFE) [45]. The results of the study indicated that fibers with oriented structures could guide axons to grow in a directional manner, while the axons of cells attached to randomly arranged fibers exhibited radial extension (Figure 5a). Furthermore, annealed P(VDF-TrFE) increased the β-phase density, enhanced the piezoelectric properties and accelerated cell growth compared to the scaffold without annealing.

Doping with different materials to increase piezoelectricity is a viable approach for neural tissue engineering. In particular, GO-based conductive nanofiber scaffolds have been found to modulate the behavior of Schwann cells, including migration, proliferation and myelin formation *in vitro*, and to promote rat pheochromocytoma 12 (PC12) cell migration, differentiation and neural regeneration *in vivo* [208]. Abzan *et al*. used non-solvent-induced phase separation to prepare PVDF/GO neural tissue engineering scaffolds, as shown in Figure 5b. The incorporation of GO into the PVDF matrix reduced the water contact angle and increased the hydrophilicity, water absorption and water flux of the scaffold. In addition, GO improved the mechanical properties of PVDF, such as toughness and strength, enhanced its piezoelectric properties, and promoted the adhesion, migration and proliferation of PC12 cells [209]. Similarly, Zhang *et al*. developed PLLA/GO scaffolds for neural regeneration [47]. The hydrophilicity of PLLA nanofiber scaffolds was increased by the introduction of hydrophilic groups such as NH_2_, COOH and OH after aminolysis and GO nanosheet coating. The scaffolds also promoted the proliferation of PC12 cells.

Genchi *et al*. fabricated P(VDF-TrFE)/BT nanoparticle films [53]. This composite piezoelectric material has been found to support the proliferation and differentiation of human neuroblastoma cells (SH-SY5Y), as determined by quantifying the expression of β3-microtubulin in the cells. Furthermore, when subjected to ultrasound stimulation, the percentage of β3-microtubulin-positive cells significantly increased, leading to Ca^2+^ transients, calcium-channel activation and increased nerve axon elongation.

Other piezoelectric polymers can also be used to make copolymers. Cheng *et al*. prepared PVDF/PCL composite scaffold materials for neural tissue engineering [210]. As a biodegradable biological material, PCL is nontoxic, and blending PCL and PVDF can increase the biocompatibility and biodegradability of the scaffold. With increasing PVDF content, the cell viability and proliferation capacity increased significantly (Figure 5c). In *in vivo* experiments in rats, the composites stimulated the growth of Schwann cells, the formation of myelin sheaths, the regeneration of axons and vasculogenesis.

PPy and PANi, which are highly conductive polymers, have the potential to promote neuronal synapse growth in neural tissue engineering applications, particularly in bioscaffolds fabricated by coblending with PLLA [211]. Zou *et al*. fabricated directed conductive PPy/PLLA fiber membranes through electrostatic spinning, and electrical stimulation of PC12 cells cultured on the fiber membrane *in vitro* increased the growth of neuronal synapses (Figure 5d) [212]. Zhou *et al*. developed a bioactive scaffold suitable for electrical stimulation by coating various ECM components, such as laminin, fibronectin and collagen, on the surface of PPy/PLLA to promote neuronal cell adhesion, growth and extension (Figure 5e) [213]. Chen *et al*. prepared GO/PPy/PLLA composite membranes through the electrochemical deposition of PPy/PLLA composites doped with GO, and the presence of GO increased the electrical conductivity and tensile strength of the material [214,215]. *In vivo* experiments on sciatic nerve repair in Sprague Dawley (SD) rats showed that the composites induced muscle regeneration and neurosynaptic regeneration (Figure 5f). Furthermore, Prabhakaran *et al*. fabricated PLLA/PANi blended composite polymer nanofiber scaffolds using electrostatic spinning, and electrical stimulation provided by this blended polymer also promoted nerve cell synapse elongation [216].

#### Skin tissue engineering

The skin, the largest organ of the human body, contains an abundance of sensory nerves that respond to external pressure signals and exhibit piezoelectric behavior by converting them into electrical signals. The potential of electrical stimulation for augmenting blood flow, promoting granulation tissue growth, promoting fibroblast proliferation and collagen deposition, inducing epidermal cell migration, and facilitating epithelial cell tissue growth has been extensively investigated [217,218].

**Figure 6:**
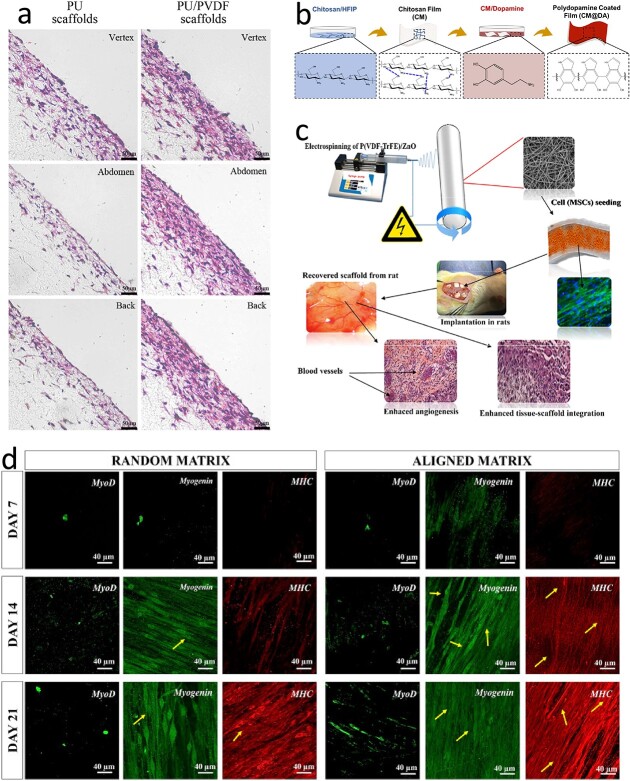
Piezoelectric materials for other tissue regeneration. (**a**) H&E staining of the PU/PVDF scaffolds and the PU scaffolds explanted from the vertex, abdomen and back of SD rats (scale bar 50 μm). (Reprinted with permission from ref. [41] © 2012 Elsevier B.V.) (**b**) Schematic illustration of the fabrication of pure chitosan film (CM) and polydopamine coated chitosan film (CM@DA). The blue dashed lines represent the inter-molecular hydrogen bonds. (Reprinted with permission from ref. [220] © 2020 Elsevier B.V.) (**c**) Schematic representation of the production of electrospun P(VDF-TrFE)/ZnO nanocomposite scaffolds, hMSC seeding of scaffolds and implantation in rats (figures are not to scale). (Reprinted with permission from ref. [31] © 2017 Springer Nature Limited.) (**d**) MyoD, myogenin and MHC immunohistochemistry staining confocal microscopy images of C2C12 cells on random and aligned poly-3-hydroxybutyrate/poly-β-alanine fibrous matrices on predetermined days of cell culture (scale bar: 40 μm). Yellow arrows indicate myofibrils. MyoD (green) antigen was detected in the cell nucleus, myogenin (green) and MHC (red) antigens were detected in cytoskeleton and thick filaments, respectively. [244]. *H&E* hematoxylin-eosin, *SD* Sprague Dawley, *MyoD* Myoblast determination protein 1, *myogenin* myogenic factor 4, *MHC* myosin heavy chain, *PU* Polyurethane, *PVDF* polyvinylidene fluoride, *HFIP* 1,1,1,3,3,3-hexafluoro-2-propanol, *P(VDF-TrFE)* poly(vinylidene-fluoride-trifluoroethylene), *ZnO* zinc oxide, *hMSC* human mesenchymal stem cells

Guo *et al*. fabricated PU/PVDF coblended scaffolds using the electrostatic spinning method, and the scaffold exhibited favorable mechanical and piezoelectric properties [41]. An *in vitro* activity assay of the scaffold material revealed that fibroblasts adhered to the scaffold surface and underwent migration and proliferation. Moreover, the presence of PVDF has been found to increase the expression of collagen, elastin and fibronectin. *In vivo* transplantation experiments in SD rats demonstrated the biocompatibility of the PU/PVDF scaffold, which elicited a greater degree of fibrosis and faster wound healing than did the PU scaffold alone (Figure 6a).

Goonoo *et al*. developed a fibrous pad composed of polydioxanone (PDX)/poly(hydroxybutyrate-co-valerate) (PHBV) for promoting skin regeneration [219]. PDX provided mechanical properties comparable to those of elastin and collagen, while PHBV exhibited piezoelectric properties. By varying the ratio of the two polymers, the wettability, surface charge and mechanical properties of the scaffold were adjusted to tune the ability of the scaffold to support cell adhesion, proliferation and migration, and the 20/80 scaffold performed best. The PDX/PHBV coblended scaffold increased fibroblast proliferation and migration, reduced macrophage-induced inflammation and accelerated scar-free wound regeneration *in vivo*.

Bhang *et al*. created ZnO nanosheets for skin wounds, and the resulting electrical stimulation promoted cell migration and growth, as well as fibronectin synthesis [29]. Chen *et al*. developed a bifunctional film with piezoelectric and photothermal properties, named CM@DA, which was coated with polydopamine on a chitosan film to combine electrical stimulation with thermal therapy for wound healing (Figure 6B) [220]. CM@DA generates voltage under mechanical pressure and exhibits photothermal effects under near-infrared light irradiation. The material has been found to increase cell proliferation, migration, angiogenesis and collagen deposition, thereby promoting wound healing.

In addition to nanofibers, hydrogel dressings have been found to have a pronounced effect on the healing of damaged wounds in skin tissue engineering [221–227]. Hydrogels possess a high water content and exhibit liquid-like fluidity, allowing them to change shape and move autonomously under natural conditions. These materials are biocompatible and can serve as drug carriers, delivering drugs to the wound site [228,229].

Electrically conductive hydrogels that incorporate new functional nanomaterials hold significant promise for electronic skins and for personalized medical monitoring, particularly in the field of wearable strain sensors [230,231]. Electrically conductive hydrogels include a hydrogel component that provides a highly hydrated environment and a conductive material component that imparts electrical conductivity. Recent studies have shown that conductive hydrogel dressings possess hemostatic, antimicrobial and anti-inflammatory properties, and promote wound healing [232,233]. Additionally, electrical stimulation has been found to promote cell migration, angiogenesis, cell proliferation and collagen deposition [234]. Hermenegildo *et al*. prepared CoFe_2_O_4_/gellan gum methacrylic acid/PVDF hydrogel scaffolds [235] with a porous 3D structure, high biocompatibility and an appropriate mechanical/electrodynamic response when triggered by an applied magnetic field, which will provide a basis for new tissue regeneration strategies.

Liang *et al*. used 3D printing technology to manufacture a PVDF/sodium alginate (SA) piezoelectric hydrogel scaffold (ZPFSA) modified with ZnO nanoparticles [236]. PVDF continuously senses pressure and produces an electrical signal. The SA serves as the support body of the stent network. ZnO nanoparticles are uniformly distributed in the scaffold network to modify PVDF, which has stable hydrophilic polarization and good antibacterial properties. ZPFSA (containing 0.5% ZnO nanoparticles) scaffolds modulate wound healing cascade events, including cell migration, angiogenesis, collagen remodeling and the expression of related growth factors. Du *et al*. developed a bioinspired hybrid patch, known as HPSP, with self-adhesive and piezoelectric nanogenerators for skin wound healing [237]. The patch comprises a mussel-inspired PDA-polyacrylamide hydrogel matrix and a piezoelectric nanogenerator based on oriented electrospun PVDF nanofibers. The resulting *in situ*-formed hydrogels are biomimetic materials that mimic the strong wet adhesion capability observed in mussels and exhibit strong resistance to wet adhesion [238]. The prepared HPSP can adhere to the wound site and generate local low-frequency pulsed voltage induced by biomechanical energy, promoting fibroblast proliferation and migration while simultaneously effectively increasing collagen deposition, angiogenesis and re-epithelialization *in vivo* by elevating the expression of key growth factors. Li *et al*. reported an electroactive hydrogel consisting of polyacrylonitrile, acrylamide, styrene sulfate and PVDF (PAAN-PVDF) that exhibits high stretchability and skin-like ductility [239]. By introducing acrylonitrile and PVDF, the strength of the composite hydrogel is increased, and its piezoelectric properties are enhanced by dipole–dipole interactions. Moreover, the Young’s modulus is comparable to that of human skin. This work provides innovative strategies for the prevention and early treatment of pressure injuries/pressure ulcers. In a recent study, BT nanotubes and polyacrylamide hydrogels were combined to create a multimodal biomechanical sensor capable of detecting human motion, pressure and curvature [240]. This study highlights the potential for integrating BT piezoelectric ceramics into hydrogels for potential use in tissue engineering applications.

#### Muscle tissue engineering

Human skeletal muscle is widely distributed and plays a crucial role in the control of strength and body movement in the human body. It is characterized by extensive vascular, lymphatic and neural networks. As a soft tissue, muscle tissue is very vulnerable to injury. After minor injury, muscle satellite cells or MSCs orchestrate the self-repair process. The application of piezoelectric materials in muscle tissue engineering is focused on simulating the bioelectrical signals generated by muscles during movement. The piezoelectric and dielectric properties of these materials allow them to generate microelectric fields during muscle contraction and relaxation, which can stimulate muscle cell proliferation and muscle fiber regeneration [241–243]. These materials must possess sufficient mechanical properties to maintain structure and function during the dynamic movement of muscles.

Martins *et al*. investigated the influence of PVDF with varying polarity and fiber morphology on myogenic cell adhesion and proliferation [42]. The results demonstrated that oriented electrospun PVDF fibers facilitated directed cell growth and regenerated muscle tissue, exhibiting physiological activity in response to electrical stimulation.

Konuk Tokak *et al*. fabricated poly-3-hydroxybutyrate (P3HB)/poly-β-alanine (PBA) fibrous tissue scaffolds using electrostatic spinning [244]. The addition of PBA and the alignment of the fibers reduced the crystallinity and brittleness of the P3HB matrix. Both random and oriented scaffolds were prepared, and the tensile strength and elastic modulus of the oriented fiber scaffolds were found to be significantly greater than those of the random fibers. C2C12 myogenic cells grew more regularly on the oriented fiber scaffolds and exhibited a similar structure to natural muscle tissue. Myoblast determination protein 1, myostatin and myosin heavy chain are the principal myogenic factors that control myogenic differentiation, and the detection of the gene expression of these factors showed that the oriented fibers effectively supported myogenic differentiation (Figure 6d). Thus, the P3HB/PBA fiber scaffold has potential for mimicking the natural regeneration of skeletal muscle tissue. Blanquer *et al*. demonstrated that both smooth muscle cells and skeletal muscle cells can adhere, proliferate and migrate on ZnO nanogenerators [30].

#### Cardiovascular tissue engineering

The myocardium is a complex tissue composed of multiple cell types, mainly cardiomyocytes and fibroblasts. Cardiomyocytes generate electrical signals through ion-flow channels in the cell membrane [245] to control the rhythm and regularity of the heartbeat by coordinating contraction and relaxation. The proper functioning of this system ensures that the heart pushes blood to the arterial system during contraction and allows the cardiac chambers to fill during relaxation. In addition, myocardial electrical signaling plays a decisive role in maintaining the rhythm and heart rate of the heart, ensuring the stability and effectiveness of the heart’s pumping function. Any abnormality that affects myocardial electrical signaling can lead to serious heart problems such as arrhythmia. Fibroblasts are responsible for maintaining the ECM, which contains collagen and elastin, providing structural support and cell alignment for the myocardium [245]. Cardiomyocytes are connected to each other through interstitial junctions to form a coordinated structure that ensures efficient pumping of blood by the heart and maintains normal blood circulation in the body.

Cardiac electrical-signal conduction disorders can lead to arrhythmia, heartbeat incoordination or other problems and, in severe cases, may even lead to cardiac arrest and other life-threatening conditions. To solve this problem, researchers have attempted to develop piezoelectric materials to restore the systolic function of the heart [246,247]. The development and application of these materials is expected to provide new treatment options for patients with heart disease, improve quality of life and reduce the serious consequences caused by disturbances in the electrical conduction of the heart.

In a study conducted by Hitscherich *et al*., the effects of P(VDF-TrFE) on mouse embryonic stem cell-differentiated cardiomyocytes and endothelial cells were investigated [248]. The results showed that both mouse embryonic stem cell-differentiated cardiomyocytes and endothelial cells can adhere to P(VDF-TrFE) nanofibers and exhibit good contractile function, as evidenced by the detection of cardiomyocyte-specific proteins, such as myosin heavy chain, cardiac troponin T and gap junction protein 43 (CX43). Additionally, endothelial cell-specific proteins, including platelet endothelial cell adhesion molecules and endothelial nitric oxide synthase, were detected, indicating that the differentiated cells were able to function. This study demonstrated the feasibility of using P(VDF-TrFE) as a cardiovascular scaffold. Adadi *et al*. also developed a P(VDF-TrFE)-based fibrous scaffold that can serve as both a biological scaffold and a sensor for myocardial regeneration and systolic function measurement [249]. They demonstrated the differentiation of human induced pluripotent stem cells into cardiomyocytes, as well as their long-term culture and maturation on P(VDF-TrFE) scaffolds.

Augustine *et al*. employed P(VDF-TrFE)-doped ZnO nanoparticles to produce a P(VDF-TrFE)/ZnO composite piezoelectric material (Figure 6c) with remarkable cytocompatibility and hemocompatibility [31]. The incorporation of ZnO particles into the composite led to the upregulation of growth factors, including fibroblast growth factor and vascular endothelial growth factor, which effectively promoted cell adhesion, migration, proliferation and angiogenesis [250].

BG is an effective biomaterial for inducing angiogenesis [251,252]. Li *et al*. fabricated an excellent biocompatible piezoelectric scaffold by combining KNN and BG. By analyzing the expression of endothelial nitric oxide synthase, intracellular calcium levels and changes in cell membrane potential, it was concluded that the scaffold regulates angiogenesis by activating the endothelial nitric oxide synthase/NO signaling pathway under the synergistic effect of electrical stimulation and activating ions, further illuminating the potential mechanisms by which angiogenesis can be promoted [253].

### Challenges and future perspectives

**Figure 7:**
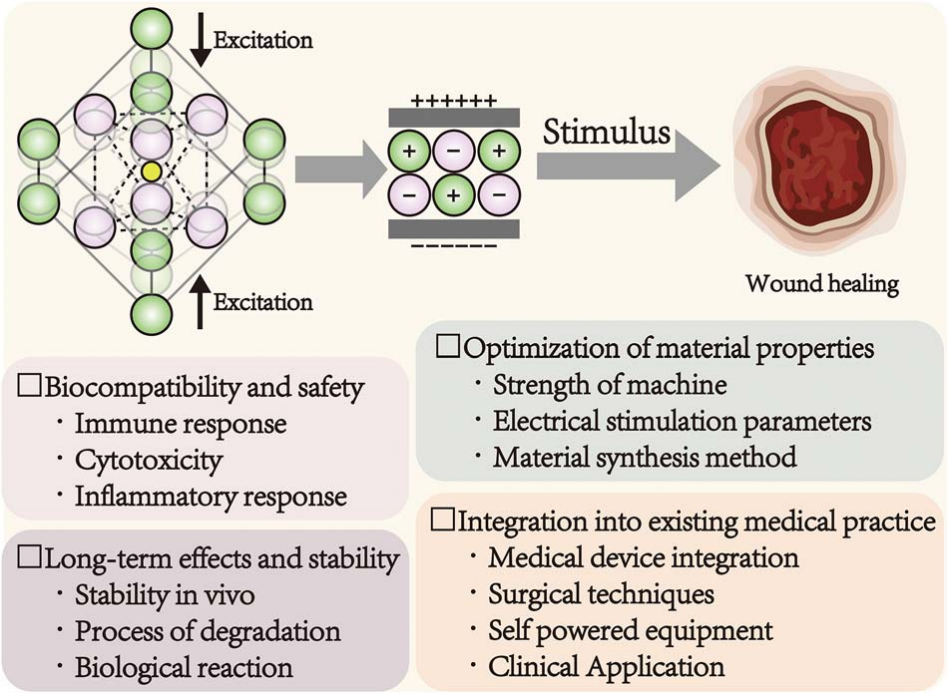
Challenges in the development of piezoelectric materials for tissue engineering

This paper presents a comprehensive review of the principles, classification and application of piezoelectric materials in tissue regeneration. Piezoelectric scaffolds possess the unique ability to generate electrical signals without external power sources or implantable batteries and can influence host cell behavior and facilitate the repair and regeneration of damaged tissue. A range of piezoelectric materials, including polymers such as PVDF, P(VDF-TrFE), PLLA and PHB, ceramics such as BT, ZnO and HA, and composites such as PVDF/BT, have been extensively utilized for this purpose. Notably, composite piezoelectric materials such as PVDF/BT exhibit excellent mechanical properties and satisfactory piezoelectric coefficients in studies involving bone, nerve and skin tissue regeneration. However, despite these promising results, the application of piezoelectric materials in tissue engineering is still in its infancy and faces multiple challenges. Future research will focus on the following four directions, as shown in Figure 7.

#### Biocompatibility and safety

The key task in optimizing the application of piezoelectric materials is to maintain piezoelectric properties while increasing biocompatibility and reducing potential toxicity. This challenge requires addressing multiple aspects to ensure that piezoelectric materials can function safely and effectively when in contact with an organism. The priority is to increase biocompatibility by promoting cell adhesion, growth and functional properties through precise surface modifications and by decreasing the risk of adverse effects. Moreover, to prevent coagulation, hemolysis and immune responses, interactions with the blood and immune systems need to be considered and potentially adjusted by the modulation of material surface properties. In addition, future research directions should include the development of environmentally sustainable and nontoxic piezoelectric materials, as well as the adoption of advanced manufacturing techniques such as 3D printing, to simultaneously increase the efficacy of specific applications and reduce resource waste.

#### Long-term effects and stability

The key challenge is to determine the balance between the degradation rate of piezoelectric scaffolds and the kinetics of tissue repair. This task requires finding the ideal combination of functionality and biocompatibility of the material to ensure that cells can grow in a stable piezoelectric environment and to prevent the material from degrading too rapidly or too slowly and thus interfering with the repair process. Using computer-aided design and simulation technology, the degradation rate and piezoelectric effect of materials can be precisely regulated, and personalized scaffold design can be performed to provide improved support for tissue repair.

#### Optimization of material properties

Different tissue types, such as bone, nerve, tendon and skin, have unique structures and morphologies that affect tissue regeneration and repair. To effectively apply nonendothelial electrical stimulation to promote tissue regeneration and repair, the role of endogenous bioelectricity in tissue development and repair needs to be understood. Examining how endogenous bioelectricity affects cell proliferation, differentiation, migration and synthesis of ECM can help us identify specific cellular and biological processes to be targeted by nonendogenous electrical stimulation. For different tissues, such as bones, nerves, tendons and skin, piezoelectric materials that mimic the structure and function of natural tissues need to be developed and customized. For example, bone regeneration requires materials that mimic the properties of bone, while nerve regeneration requires an environment that supports nerve cell growth and orientation. Individualized design and rigorous experimental validation are essential to ensure the effective application of piezoelectric materials in the regeneration of specific tissues. In addition, interdisciplinary collaboration can provide a more holistic perspective, helping us optimize the parameters of electrical stimulation, select appropriate biomaterials and vectors, and tailor the mode and timing of electrical stimulation to achieve optimal therapeutic outcomes.

#### Integration into existing medical practice

The application of piezoelectric materials in implantable medical electronic devices can support multiple critical functions, such as signal detection, data acquisition and monitoring. Improvements in these areas have broad implications for medical research and clinical practice, particularly in chronic disease management, individualized medicine and rehabilitation. The electrical activity of piezoelectric materials enables their use as sensors for weak biological signals, which can provide valuable information on physiological parameters to aid in health monitoring and therapeutic evaluation. This trend reflects the rapid development of medical electronics and the need for versatility.

In general, the electroactive properties of piezoelectric materials provide new tools for monitoring and regulating cell activities and tissue regeneration, and these materials are expected to drive innovations in personalized medicine, therapeutic monitoring and rehabilitation. However, challenges such as achieving suitable biocompatibility and material stability and matching to different tissues will require intensive research and innovation. Balancing piezoelectric properties and biocompatibility and adjusting degradation rates to suit the repair kinetics of a particular tissue are critical tasks during material design and fabrication. To promote sustainability, interdisciplinary collaboration will become particularly important, and collaboration between scientists and engineers will help to achieve a deeper understanding of the behavior of piezoelectric materials in different biological environments. In addition, innovative manufacturing techniques such as 3D printing and nanofabrication will support the fabrication of customized, high-efficiency piezoelectric materials. Together, these opportunities and challenges will shape the future of piezoelectric materials in biomedicine.

## Conclusions

In summary, piezoelectric materials hold great promise for applications in tissue engineering and biomedicine, but a series of challenges must first be overcome. With unremitting effort, collaboration and innovation, we can expect rapid development in this field, bringing innovations and breakthroughs to the field of medicine and health.
